# Nirsevimab Effectiveness Against RSV‐Related Hospitalisations in Children Under 24 Months: A Test‐Negative Case–Control Study in Portugal, 2024–2025

**DOI:** 10.1111/irv.70186

**Published:** 2025-11-30

**Authors:** Vânia Gaio, Camila Henriques, Miguel Lança, Rita Marques, Raquel Marques, Marta Rodrigues, Sofia Almeida, Beatriz Sousa, Margarida Freitas, Diana Amaral, Sara Ferreira, Inês Azevedo, Madalena von Hafe, Rafaela Gonçalves, Regina Viseu, Teresa Bandeira, Carolina Constant, Madalena Malato, Inês Carvalho, Jorge Rodrigues, Margarida Farinha, Teresa Nunes, Teresa Graça, Sofia Gomez, Sara Soares, João Farela Neves, Paulo Paixão, Inês Piscalho, Ana Loureiro, Cristina Freitas, José Alves, Diana Soares, Paulo Lopes, Ausenda Machado, Raquel Guiomar, Ana Paula Rodrigues, VigiRSV Group, Ana Carolina Pinto, Liliana Macedo, Paula Mota, Maria Francisca Santos, Carlota Ferreira, Elsa Melanie dos Santos, Ana Pinto, Tomás Tinoco, Natasha Rosário, Xavier Ferreira, Susana Maia, Inês Lopes, Vânia Soares, Arminda Jorge, Patrícia Ibarzabal, Joana Leal, Inês Couto, Beatriz Lourenço, Cláudia Fernandes, Carolina Ferreira, Bruna Barbosa, Catarina Encarnação, Rosário Barreto, Ana Sofia Santos, Marisa Nunes, Raul Martins, Ana Dias Alves, Florbela Cunha, Carla Cruz, Nádia Cavaco, Pedro Fernandes da Gama, Elsa Rocha, Cláudia Calado, Bernardo Camacho, Andreia Afonso e, Fátima Pestana, Raquel Ferreira

**Affiliations:** ^1^ Department of Epidemiology National Institute of Health Doutor Ricardo Jorge Lisbon Portugal; ^2^ NOVA National School of Public Health, Public Health Research Centre, Comprehensive Health Research Center, CHRC, REAL, CCAL NOVA University Lisbon Lisbon Portugal; ^3^ Department of Infectious Diseases National Institute of Health Doutor Ricardo Jorge Lisbon Portugal; ^4^ NOVA Medical School, Faculdade de Ciências Médicas, Universidade NOVA de Lisboa Lisbon Portugal; ^5^ Unidade Local de Saúde de Almada‐Seixal Almada Portugal; ^6^ Unidade Local de Saúde da Cova da Beira Covilhã Portugal; ^7^ Unidade Local de Saúde do Alto Ave Guimarães Portugal; ^8^ Unidade Local de Saúde de São José Lisbon Portugal; ^9^ Unidade Local de Saúde de São João Porto Portugal; ^10^ Unidade Local de Saúde da Arrábida Setúbal Portugal; ^11^ Unidade Local de Saúde de Santa Maria Lisbon Portugal; ^12^ Unidade Local de Saúde do Estuário do Tejo Vila Franca de Xira Portugal; ^13^ Unidade Local de Saúde de Viseu Dão‐Lafões Viseu Portugal; ^14^ Hospital dos Lusíadas Lisbon Portugal; ^15^ Unidade Local de Saúde do Alentejo Central Évora Portugal; ^16^ Hospital da Luz Lisbon Portugal; ^17^ Unidade Local de Saúde do Algarve Faro Portugal; ^18^ Hospital Dr. Nélio Mendonça Funchal Portugal; ^19^ Unidade Local de Saúde de Gaia/Espinho Vila Nova de Gaia Portugal

**Keywords:** children, effectiveness, nirsevimab, RSV‐related hospitalisations

## Abstract

We assessed Nirsevimab effectiveness (NE) against respiratory syncytial virus (RSV)‐related hospitalisation in eligible children (< 2 years) using a test‐negative case–control design within the VigiRSV network (weeks 43/2024 to 16/2025). Among 341 participants (median age: 2 months; 91.2% without known chronic condition), 137 (40.2%) tested RSV‐positive. Adjusted NE against RSV‐related hospitalisation was 78.5% (95%CI: 59.3–89.0). Sensitivity analyses confirmed the robustness of the results. These findings support Nirsevimab's effect in a predominantly healthy infant population and contribute to informing public health decisions for RSV immunisation.

## 1 Background

Respiratory syncytial virus (RSV) is a leading cause of lower respiratory tract infections in young children, causing an estimated 3.6 million hospitalisations and 100,000 deaths globally in 2019 among children under 5 years old [1]. In Portugal, nearly 9700 RSV‐related hospitalisations occurred in 2015–2018, mostly in previously healthy infants with an estimated annual cost of €2.4 million [2]. Following disruptions to RSV seasonality during the COVID‐19 pandemic, including a summer surge in 2021 [3, 4], Portugal established a sentinel surveillance network—VigiRSV—for hospitalised children under 24 months [5].

In 2023, the European Medicines Agency approved Nirsevimab, a long‐acting monoclonal antibody, for all newborns and infants [6]. Early real‐world evidence from Spain, reported high Nirsevimab effectiveness against RSV‐related hospitalisations [7]. Portugal began universal Nirsevimab immunisation in the Autonomous Region of Madeira in the 2023/2024 season [8], with nationwide implementation in 2024/2025 [9]. This study aims to estimate the effectiveness of the monoclonal antibody **Nirsevimab** against RSV‐confirmed hospitalisations among children under 24 months during the 2024/2025 season in Portugal.

## 2 Methods

### 2.1 Setting, Study Design and Population

We conducted a test‐negative case–control study within the VigiRSV sentinel hospital network, comprising 15 hospitals across mainland Portugal and Madeira Island. Eligible participants were children under 24 months with severe acute respiratory infection (SARI), defined as the acute onset of respiratory symptoms—cough, sore throat, shortness of breath or coryza—and a clinician's judgement that the illness was due to an infection and needed of hospitalisation. Infants under 6 months presenting with apnoea or sepsis of unknown cause were also included [10]. All participants were tested for RSV using PCR or rapid antigen tests. Cases were RSV‐positive patients and controls tested negative.

The study period spanned from week 43/2024—1 week after the start of the national immunisation campaign (15 October 2024)—to Week 16/2025, when case notifications ceased. In addition to the standard VigiRSV eligibility criteria, this study included only children eligible for immunisation under national guidelines: (A) infants born between 1 August 2024, and 31 March 2025; (B) preterm infants born between 1 January and 31 July 2024, with a gestational age of up to 33 weeks and 6 days; and (C) children under 24 months as of 30 September 2024, with medical conditions associated with increased risk of severe RSV infection [9].

Exclusion criteria included hospital readmissions during the study period, maternal RSV immunisation during pregnancy, respiratory sample collection more than 7 days after symptom onset, and birth after 31 March 2025.

Data were collected by trained healthcare professionals using a standardised electronic questionnaire (REDCap) [11], based on clinical records. Information included demographics, clinical presentation, underlying conditions, immunisation history (including maternal RSV immunisation), laboratory test results, Intensive Care Unit (ICU) admission and oxygen support and hospital outcomes.

### 2.2 Definitions of Exposure and Outcome

The exposure of interest was immunisation with Nirsevimab administered at least 2 days before symptom onset. Immunisation status and dates were validated by hospital teams using the national vaccination registry. The outcome was RSV‐related hospitalisation, confirmed by laboratory testing (reverse transcription polymerase chain reaction (RT‐PCR) or rapid antigen test).

### 2.3 Data Analysis

Differences in categorical variables (proportions) between cases and controls were assessed using Pearson's Chi‐squared test. Continuous variables were compared using the Wilcoxon rank‐sum test. Nirsevimab effectiveness (NE) was estimated as NE = (1—OR) × 100%, where OR is the odds ratio for Nirsevimab immunisation among cases versus controls. ORs were obtained using logistic regression adjusted for age group (< 1, 1–2, 3–5, 6–11 and 12–23 months), sex (male/female), month of symptom onset (October 2024 to April 2025), the presence of chronic condition, prematurity and low birthweight. Sensitivity analyses included: (1) restricting to participants tested by RT‐PCR only; (2) considering immunisation at least 7 days before symptom onset; and (3) excluding data from Madeira. All statistical analyses were performed using R version 4.0.5 (R Foundation for Statistical Computing, Vienna, Austria [12]).

## 3 Results

### 3.1 Participant's Characteristics

**FIGURE 1:**
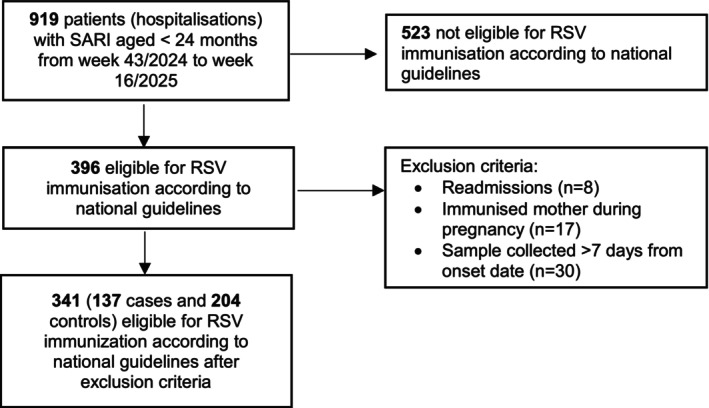
Flow chart of participants selected for the study.

Between Week 43/2024 and Week 16/2025, 919 hospitalised children under 24 months with SARI were reported through the VigiRSV network. Of these, 396 were eligible for RSV immunisation according to national guidelines. After applying exclusion criteria, 341 children were included in the analysis: 137 RSV‐positive cases and 204 RSV‐negative controls (Figure 1); 98% of children were experiencing their first complete RSV season.

**FIGURE 2:**
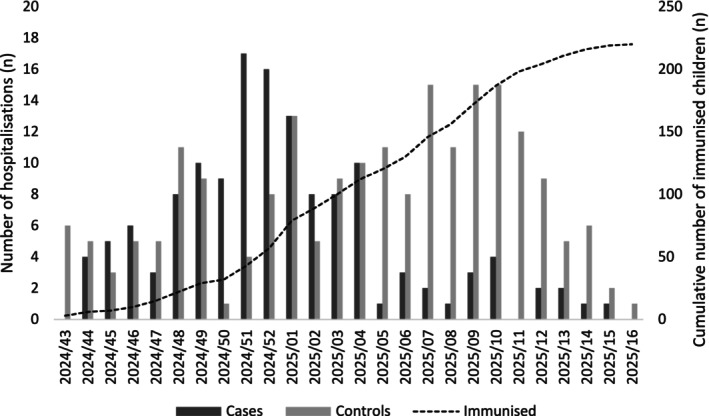
Distribution of cases and controls on the epidemic curve, considering the study period between Week 43/2024 and Week 16/2025.

The distribution of cases and controls, along with the cumulative number of immunised children, shows that cases were predominantly selected from weeks with low immunisation coverage. In contrast, controls were mostly selected from weeks with higher immunisation levels, up until Week 16 of 2025, when an immunisation coverage of 64.5% (*n* = 220/341) was achieved (Figure 2).

Among the 341 participants, cases (*n* = 137) and controls (*n* = 204) were similar in terms of sex, prematurity, low birth weight, chronic conditions and median days between immunisation and symptom onset (*p*‐values > 0.05). A significant difference was observed regarding age group and immunisation status, with a higher proportion of controls (77.9%) having received immunisation compared to cases (44.5%) (*p* < 0.01) (Table 1).

**TABLE 1:** Cases and controls characterization.

Characteristics	Total (*N* = 341)	Cases (*N* = 137)	Controls (*N* = 204)	*p*
Age group *n* = 341				**0.01**
< 1 month	60 (17.6%)	15 (11.0%)	45 (22.1%)	
1–2 months	143 (41.9%)	65 (47.4%)	78 (38.2%)	
3–5 months	76 (22.3%)	33 (24.1%)	43 (21.1%)	
6–11 months	41 (12.0%)	20 (14.6%)	21 (10.3%)	
12–23 months	21 (6.2%)	4 (2.9%)	17 (8.3%)	
Age (median, IQR)—months *n* = 341	2 (1–4)	2 (1–3)	2 (1–4)	0.28
Sex (% males) *n* = 341	224 (65.7%)	88 (64.2%)	136 (66.7%)	0.73
Prematurity* (%) *n* = 322	73 (22.7%)	28 (21.4%)	45 (23.6%)	0.92
Low birth weight** (%) *n* = 310	68 (21.9%)	27 (21.3%)	41 (22.4%)	0.75
Chronic condition*** (%) *n* = 341	30 (8.8%)	11 (8.0%)	19 (9.3%)	0.83
Nirsevimab immunisation (%) *n* = 341	220 (64.5%)	61 (44.5%)	159 (77.9%)	**< 0.01**
Days between immunisation and symptoms onset (median, IQR)‐days	48.0 (25.8–76.3)	49.0 (34.0–59.0)	47.0 (17.0–79.0)	0.73

* Prematurity was defined as gestational age < 37 weeks.

** Low birth weight was defined as birth weight < 2500 g.

*** Chronic conditions included the following: congenital heart disease, velocardiofacial syndrome, chronic lung disease, trisomy 21, immunodeficiency and neuromuscular disease.

### 3.2 Nirsevimab Effectiveness Against RSV‐Related Hospitalisations

In the primary analysis (*n* = 341), the adjusted Nirsevimab effectiveness against RSV‐related hospitalisations was 78.5% (95% CI: 59.3–89.0) (Table 2). Sensitivity analyses revealed that among PCR‐tested participants (*n* = 255), Nirsevimab effectiveness was 82.0% (95% CI: 62.0–91.8) (Table 2).

**TABLE 2:** Nirsevimab effectiveness against RSV‐related hospitalisations among children under 24 months during the 2024/2025 RSV season, in Portugal.

	Cases nirsevimab immunisation (n/n, %)	Controls nirsevimab immunisation (n/n, %)	NE (%) crude (95% CI)	NE (%) adjusted (95% CI)*
Primary analysis (*n* = 341)	61/137, 44.5%	159/204, 77.9%	77.3 (63.8–86.0)	78.5 (59.3–89.0)
Sensitivity analysis				
Only PCR‐tested (*n* = 255)	46/101, 45.5%	126/154, 81.8%	81.4 (67.5–89.6)	82.0 (62.0–91.8)
Immunisation at least 7 days before the onset date instead of 2 days (*n* = 341)	63/137, 46.6%	161/204, 78.9%	77.3 (63.6–86.0)	76.2 (55.2–87.7)
Without Madeira data (*n* = 329)	60/134, 44.8%	153/195, 78.5%	77.7 (64.2–86.4)	79.1 (59.5–89.6)

* adjusted by age group (<, 1–2, 3–5, 6–11, and 12–23 months), sex (male/female), month of infection (oct24/nov24/dez24/jan25/fev25/mar25/apr25), chronic condition (yes/no), prematurity (yes/no), low birthweight (yes/no).

## 4 Discussion and Conclusions

In 2024/2025, the effectiveness of nirsevimab against RSV‐related hospitalisation estimated in Portugal in the target group for immunisation was 78.5% (95% CI: 59.3%–89.0%). This estimate is consistent with those reported in studies conducted in other countries, varying between 73.3% and 98%, indicating high effectiveness of the monoclonal antibody in preventing hospitalisation due to RSV‐associated acute respiratory infection [7, 13, 14, 15, 16, 17, 18].

Despite methodological differences across studies and variations in the target populations for immunisation between countries, all observational studies published to date during the first year of nirsevimab implementation, reported reductions in RSV‐related hospitalisations [19]. Some also demonstrated a reduction in RSV bronchiolitis attended at primary care [20], in intensive care unit (ICU) admissions [14, 16, 17, 18] and emergency department visits due to RSV infection among the immunised population [15].

According to the VigiRSV surveillance data during the 2024/2025 season, we observed a reduction in the incidence of RSV‐associated hospitalisations in children under 24 months of age, as well as a decrease in the proportion of RSV cases in children admitted to ICU or requiring ventilation (5% in 2024/2025 vs. 13% in 2022/2023 and 10% in 2023/2024) [21, 22]. A reduction in the proportion of RSV cases in children aged less than 3 months old compared to previous seasons (21% vs. 57% in 2022/2023 and 46% in 2023/2024) was also observed, but no differences in health risk conditions were found [21, 22]. It should be noted that Nirsevimab was included in the immunisation programme in one Portuguese region during the 2023/2024 season [5], but only in 2024/2025 was it recommended all across the country. Nevertheless, Nirsevimab effectiveness for regions recommending it for the first year was similar to the overall results.

Sensitivity analysis revealed similar results when restricting to those tested with RT‐PCR or those immunised for more than 7 days before symptom onset, indicating a low risk of outcome and exposure misclassification bias in our estimates.

While it was not an objective of this study, we found a Nirsevimab coverage of 77.9% among controls, which is lower than the coverage of other vaccines of the National Immunisation Plan for the same age group. Our study comprised hospitalised children, who have different risk profiles compared with nonhospitalised children of the same age. However, higher coverages in studies from other countries with hospitalised patients had been reported [20]. Having no official data on Nirsevimab coverage during the season prevents us from comparing it to identify a possible bias on patient recruitment. As we did not investigate the reasons why eligible patients were not immunised, it could be useful to investigate factors associated with nonimmunisation status during this first national campaign, to tailor next interventions for the future implementation of the programme.

Despite the high Nirsevimab effectiveness in our study and in other studies as well, the continuous surveillance of RSV and other respiratory pathogens is highly recommended to monitor long‐term impacts [20] and possible changes in circulating viruses. For example, in Spain, during the 2023/2024 season, a 30% reduction in RSV bronchiolitis was observed in immunised children compared with eligible but nonimmunised children. However, an increase in hospitalised cases of bronchiolitis due to human metapneumovirus and rhinovirus was reported towards the end of the season [23]. These findings might not be related to the decrease of RSV infections but reinforce the importance of an integrated year‐round surveillance of respiratory pathogens, encompassing epidemiologic, virological and genomic data.

This study has several limitations. First, as a test‐negative case–control design, it is inherently observational and may be subject to residual confounding, despite adjustment for key variables such as prematurity, low birth weight and chronic conditions. Second, our sample included 341 children recruited through a surveillance network of 15 hospitals across the country, which may not fully represent all hospitalised children under 24 months in Portugal. Third, information on Nirsevimab coverage in the general population was unavailable, limiting our ability to assess potential selection bias. Finally, the study period covered only the first national season of Nirsevimab implementation, which may affect the generalisability of findings to future seasons as vaccine coverage and RSV circulation evolve.

## Author Contributions

**Vânia Gaio:** conceptualization, writing – original draft, methodology, validation, writing – review and editing, formal analysis, investigation. **Camila Henriques:** validation, writing – review and editing, conceptualization. **Miguel Lança:** conceptualization, validation, writing – review and editing. **Rita Marques:** validation, writing – review and editing, validation, writing – review and editing. **Raquel Marques:** validation, writing – review and editing, validation, writing – review and editing. **Marta Rodrigues:** validation, writing – review and editing, validation, writing – review and editing, supervision, project administration, conceptualization, investigation, writing – review and editing, writing – original draft, methodology. **Sofia Almeida:** validation, writing – review and editing. **Beatriz Sousa:** validation, writing – review and editing. **Margarida Freitas:** validation, writing – review and editing, validation, writing – review and editing. **Diana Amaral:** validation, writing – review and editing. **Sara Ferreira:** validation, writing – review and editing. **Inês Azevedo:** validation, writing – review and editing. **Madalena von Hafe:** validation, writing – review and editing. **Rafaela Gonçalves:** validation, writing – review and editing. **Regina Viseu:** validation, writing – review and editing. **Teresa Bandeira:** validation, writing – review and editing. **Carolina Constant:** validation, writing – review and editing. **Madalena Malato:** validation, writing – review and editing. **Inês Carvalho:** validation, writing – review and editing. **Jorge Rodrigues:** validation, writing – review and editing, validation, writing – review and editing, supervision, project administration, conceptualization, investigation, writing – review and editing, writing – original draft, methodology. **Margarida Farinha:** validation, writing – review and editing. **Teresa Nunes:** validation, writing – review and editing. **Teresa Graça:** validation, writing – review and editing. **Sofia Gomez:** validation, writing – review and editing. **Sara Soares:** validation, writing – review and editing, validation, writing – review and editing. **João Farela Neves:** validation, writing – review and editing. **Paulo Paixão:** validation, writing – review and editing. **Inês Piscalho:** validation, writing – review and editing. **Ana Loureiro:** validation, writing – review and editing. **Cristina Freitas:** validation, writing – review and editing, validation, writing – review and editing. **José Alves:** validation, writing – review and editing. **Diana Soares:** validation, writing – review and editing, validation, writing – review and editing. **Paulo Lopes:** validation, writing – review and editing. **Ausenda Machado:** writing – review and editing, conceptualization, validation. **Raquel Guiomar:** conceptualization, validation, writing – review and editing. **Ana Paula Rodrigues:** validation, writing – review and editing, validation, writing – review and editing, supervision, project administration, conceptualization, investigation, writing – review and editing, writing – original draft, methodology. **VigiRSV Group:** writing – review and editing, validation.

## Ethics Statement

The study was approved by the Ethics Committee of the Portuguese National Health Institute Doutor Ricardo Jorge (reference number 160/2024). Written informed consent was obtained from participants' legal guardians. All data were pseudonymised; reidentification keys were held exclusively by the attending physician responsible for recruitment and data collection.

## Conflicts of Interest

The authors declare no conflicts of interest.

## Data Availability

Data are available upon reasonable request and in accordance with national data sharing policy.
